# The Fundamental Role of Linkage Uncertainty in Epidemiological Analysis of Big Data

**DOI:** 10.23889/ijpds.v9i5.2900

**Published:** 2024-09-18

**Authors:** Jacob Bor, Evelyn Lauren

**Affiliations:** 1 Department of Global Health, Boston University School of Public Health; 2 Department of Epidemiology, Boston University School of Public Health; 3 Health Economics and Epidemiology Research Office; 4 Africa Health Research Institute; 5 Department of Biostatistics, Boston University School of Public Health

## Introduction

Epidemiologists increasingly work with linked “big data”. Uncertainty in record linkage may lead to biased inferences but is often overlooked. We evaluate the impact of linkage uncertainty on statistical inference in linked big data.

## Methods

We developed a graphical framework for describing linkage uncertainty when linking multiple representations of the same entity, applied to de-identified data from South Africa’s national laboratory database. Through simulation, we systematically introduced linkage errors and measured their impact on overall accuracy (sensitivity, positive predictive value (PPV)). We evaluate how linkage errors affect bias and variance in point estimates for a hypothetical parameter of interest in clinical epidemiology: 24-month retention in care for HIV patients. We compare the roles of sampling error vs. linkage error as fundamental sources of uncertainty in datasets of varying sizes.

## Results

We simulated a population of 14,393 HIV patients, with a “true” 24-month retention of 38.7%. There were 338,056 true links. Introducing 4,200 false links reduced PPV by 5%. Removing 21,500 existing links decreased sensitivity by 5%. From 10 simulation runs, a 95% sensitivity led, on average, to a 7.4% overestimate in entries to care and a 2.2% (range: 2.1-2.4%) underestimate in 24-month retention. A 95% PPV resulted, on average, in a 7.5% underestimate in entries to care and a 1.8% (range: 1.5-2.0%) overestimate in 24-month retention.

## Conclusion

We observe that in a large sample, linkage uncertainty minimally impacts variance in point estimates but has a potentially large influence on the magnitude and direction, distinguishing it from typical sampling errors.

